# HLA-G regulation through trogocytosis: intercellular membrane transfer mechanisms and immune dysregulation in Systemic Lupus Erythematosus

**DOI:** 10.3389/fcell.2025.1664622

**Published:** 2025-11-12

**Authors:** Abhibroto Karmakar, Uma Kumar, Rachana Kamath, Hargurdas Singh, Mukhyaprana M. Prabhu, Subhradip Karmakar

**Affiliations:** 1 Department of General Medicine, Kasturba Medical College, Manipal, Manipal Academy Higher Education, Manipal, India; 2 Department of Biochemistry, All India Institute of Medical Sciences New Delhi, New Delhi, India; 3 Department of Rheumatology, All India Institute of Medical Sciences New Delhi, New Delhi, India; 4 Department of Internal Medicine, Sri Guru Ram Das Institute of Medical Sciences and Research, Sri Amritsar, India

**Keywords:** SLE - systemic lupus erythematosus, HLA-G, trogocytosis, type I interferon signalling, immune checkpoint modulation, biomarker, immunomodulatory, genetic

## Abstract

Systemic lupus erythematosus (SLE) is a complex autoimmune disorder marked by dysregulated humoral immunity, autoantibody production against nuclear and cytoplasmic antigens, and immune complex deposition that triggers widespread inflammation and tissue damage. Central to its pathogenesis are breakdowns in peripheral tolerance, aberrant T and B cell activation, and chronic type I interferon signalling, driving the disease’s heterogeneity. Emerging evidence highlights trogocytosis, a process involving the direct transfer of membrane-associated molecules between immune cells as a key immunomodulatory mechanism in autoimmunity. Through bidirectional membrane exchange, trogocytosis alters the surface receptor landscape, antigen presentation, and signalling capacity of immune cells without requiring new protein synthesis. In SLE, trogocytosis has been linked to the dysregulation of HLA-G, a non-classical MHC class I molecule with immunosuppressive properties. HLA-G interacts with inhibitory receptors such as ILT-2, ILT-4, and KIR2DL4, modulating immune responses. In SLE, aberrant HLA-G expression on immune cells, abnormal levels of soluble HLA-G in serum, and disrupted tissue-specific expression suggest impaired immune checkpoint control. These abnormalities contribute to immune dysregulation and the loss of tolerance, sustaining chronic autoimmunity. Understanding trogocytosis-mediated modulation of HLA-G may offer novel insights into disease mechanisms and therapeutic targets in SLE. This mini review examines the molecular mechanisms underlying trogocytic HLA-G transfer, characterises the dysregulated trogocytosis pathways observed in SLE patient immune cells, and evaluates the therapeutic potential of targeting these intercellular communication networks for disease management. The present review encompasses mechanistic studies of trogocytosis regulation in disease-relevant immune cell populations, analysis of HLA-G transfer kinetics and functional consequences, and assessment of pharmacological interventions that can modulate trogocytic activity to restore immune homeostasis and reduce disease activity in lupus patients, potentially offering novel precision medicine approaches for this heterogeneous autoimmune disorder.

## Introduction

Systemic lupus erythematosus (SLE) is a prototypical, chronic autoimmune disorder characterised by a profound disruption of immune tolerance, resulting in the production of a diverse repertoire of pathogenic autoantibodies (Moulton et al., 2017). This aberrant immune activation drives widespread systemic inflammation and culminates in progressive, multi-organ damage (Ameer et al., 2022). SLE pathogenesis is orchestrated by a multifactorial interplay of genetic susceptibility, environmental stimuli, and epigenetic modifications (Sutanto and Yuliasih, 2023). Genome-wide association studies (GWAS) have identified over 180 genetic loci associated with increased susceptibility to systemic lupus erythematosus (SLE), with significant enrichment in genes regulating antigen processing and presentation, type I interferon signalling, and lymphocyte activation and differentiation. These findings underscore the polygenic and immunologically complex nature of SLE (Elghzaly et al., 2022). Recent evidence suggests that trogocytosis, a process by which immune cells exchange membrane fragments and surface proteins, may represent a novel immunomodulatory mechanism in SLE pathogenesis (Miyake and Karasuyama, 2021).

Rossi et al. demonstrated that epratuzumab, a humanised anti-CD22 monoclonal antibody, induces FcγR-mediated trogocytosis to selectively strip B-cell surface markers (CD19, CD21, CD22, CD79b) without inducing global B-cell cytotoxicity. This targeted receptor removal preserves B-cell populations while reprogramming their immunophenotype and antigen-presenting function, offering a tolerogenic immunomodulatory effect. Unlike conventional B-cell depleting therapies, this strategy minimises systemic immunosuppression and supports genotype-guided precision medicine in SLE (Rossi et al., 2013). Genetic association studies have highlighted regulatory polymorphisms within both classical (HLA class I and II) and non-classical MHC loci as major contributors to systemic lupus erythematosus (SLE) susceptibility and clinical heterogeneity. Notably, HLA-G, a non-classical MHC class I molecule has emerged as a critical immunomodulator, exerting checkpoint control through its interaction with inhibitory receptors across multiple immune lineages, thereby shaping immune tolerance and autoimmunity risk (Ha et al., 2022).

Castelli et al. identified functionally relevant polymorphisms within the HLA-G promoter and 3′untranslated region (3′UTR) that regulate transcriptional activity, mRNA stability, and alternative splicing. These variants influence the expression profiles of membrane-bound and soluble HLA-G isoforms, thereby modulating the extent and specificity of immune tolerance and peripheral regulatory mechanisms (Castelli et al., 2021).

Pharmacogenomic analyses highlight the critical role of regulatory genetic variants in dictating immune phenotypes and therapeutic responsiveness within the heterogeneous SLE population. Agbakwuru and colleagues provided mechanistic evidence that trogocytosis, an intercellular membrane exchange process is modulated by such polymorphic loci, thereby influencing its efficiency and molecular specificity. These findings suggest that individual genetic backgrounds can shape trogocytic dynamics, contributing to interpatient variability in disease progression and treatment outcomes. This insight establishes a foundation for genetically informed therapeutic strategies targeting trogocytosis as a precision immunomodulatory approach in SLE (Agbakwuru and Wetzel, 2024).

Wei et al. highlighted the complex cellular crosstalk underlying immune dysregulation in systemic lupus erythematosus (SLE), emphasising the role of trogocytosis, a rapid, contact-dependent mechanism of membrane and surface molecule exchange that modulates immune cell phenotype and function (Miyake and Karasuyama, 2021; Wei et al., 2024). Unlike classical phagocytosis, trogocytosis enables selective acquisition or shedding of surface molecules, influencing intercellular communication and immune responses (Agbakwuru and Wetzel, 2024). Among these molecules, HLA-G, a non-classical MHC class I antigen, plays a pivotal role through its immunoregulatory effects suppressing NK and CD8^+^ T cell cytotoxicity, inhibiting dendritic cell maturation, and promoting regulatory T cell expansion (González et al., 2012). Aberrant expression of membrane-bound and soluble HLA-G isoforms has been observed in SLE, implicating its role in immune tolerance breakdown (Rosado et al., 2008). The regulation of HLA-G distribution via trogocytosis may constitute a novel immunomodulatory axis in SLE pathogenesis, warranting further mechanistic investigation (Feger et al., 2007; Karczmarczyk et al., 2024). HLA-G, a non-classical HLA class Ib molecule, plays a pivotal role in maintaining immune tolerance by inhibiting the cytotoxic activity of NK cells and CD8^+^ T cells, suppressing CD4^+^ T cell proliferation, and impairing dendritic cell maturation through interactions with inhibitory receptors such as ILT2, ILT4, and KIR2DL4 (Zaborek-Łyczba et al., 2021). Additionally, soluble isoforms of HLA-G contribute to immune regulation by inducing apoptosis in activated immune cells and promoting anti-inflammatory cytokine profiles (Contini et al., 2020). In patients with systemic lupus erythematosus (SLE), HLA-G expression is frequently dysregulated, characterized by reduced surface levels on monocytes and dendritic cells, impaired cytokine-induced upregulation, and deficient trogocytic acquisition by lymphocytes (Monsiváis-Urenda et al., 2011). These abnormalities may compromise tolerogenic signaling, enhance antigen presentation, and contribute to the persistence of autoreactive lymphocytes, thereby exacerbating immune dysregulation in SLE.

This mini-review critically evaluates the mechanistic role of trogocytosis in modulating HLA-G expression and its implications for systemic lupus erythematosus (SLE) pathogenesis. Trogocytosis, a contact-dependent intercellular membrane exchange process, facilitates the redistribution of immunoregulatory molecules such as HLA-G, thereby modulating immune cell phenotypes and contributing to peripheral tolerance breakdown. By integrating data from immunogenetic association studies, cellular immunology, and translational research, this review highlights how genetic variants affecting MHC regulation, immune synapse architecture, and cytoskeletal dynamics influence trogocytic efficiency and HLA-G-mediated immune checkpoint control. Dysregulated trogocytosis may alter the immunological landscape by promoting antigen spreading, impairing regulatory networks, and enabling immune escape. The review positions trogocytosis as a critical, yet underrecognized, contributor to lupus immunopathology and underscores its therapeutic potential. Targeted modulation of trogocytosis may enable selective immuno-reprogramming without global immunosuppression, offering a novel precision medicine strategy for optimising SLE management through the restoration of immune homeostasis and tolerance. This review first delineates the molecular mechanisms underlying trogocytosis, subsequently examines HLA-G mediated immune modulation in SLE, and proceeds to analyze cell-specific dysregulation, altered signaling pathways, and therapeutic prospects.

### Reframing trogocytosis: a cross-contextual mechanism for immune modulation and therapeutic modulation

Recent studies have expanded the mechanistic and translational understanding of trogocytosis across diverse immune cell types and disease contexts, revealing its dual potential as both a driver of immune dysregulation and a therapeutic lever.

#### B cells and FcγR-Mediated trogocytosis

Rossi et al. (2013) demonstrated that trogocytosis can selectively remove B cell receptor (BCR)-associated molecules, including CD22, CD19, CD21, and CD79b from autoreactive B cells via FcγR-expressing effector cells. This Fc-dependent mechanism significantly reduces surface receptor density (>80% for CD22) without inducing broad B cell depletion, offering a safer alternative to conventional therapies like rituximab. These findings support the feasibility of dual-targeting strategies that exploit trogocytosis for precision immunomodulation.

#### T and B cell crosstalk via trogocytosis

Miyake and Karasuyama (2021), highlighted trogocytosis as a rapid, bidirectional exchange of membrane-bound receptors between T and B cells, modulating antigen presentation and co-stimulatory signaling. This dynamic fine-tuning of receptor availability helps maintain immune homeostasis and self-tolerance, and may be harnessed to downregulate autoreactive B cells in autoimmune settings.

#### T cell exhaustion and ligand stripping

Nakayama et al. (2021) revealed that excessive trogocytosis between T cells and antigen-presenting cells (APCs) can strip activating ligands from APC surfaces, dampening T cell activation and promoting exhaustion, particularly in chronic infections and tumors. Therapeutic blockade of trogocytosis may preserve T cell effector functions and enhance immunotherapies targeting exhausted phenotypes.

#### Antigen modulation in B cell malignancies

Lindorfer and Taylor (2022) described FcγR-mediated trogocytosis as a mechanism of “antigen shaving,” wherein CD20-antibody complexes are removed from malignant B cells, reducing the efficacy of anti-CD20 monoclonal antibodies. These findings underscore the need for Fc-engineered or bispecific antibodies that resist trogocytic clearance and maintain cytotoxic potency.

#### CAR-T and CAR-NK cell dysfunction

Ramezani et al. (2023) reported that CAR-T and CAR-NK cells can acquire tumor antigens and inhibitory ligands (e.g., PD-L1) via trogocytosis, leading to premature dysfunction and reduced cytotoxicity. Combining CAR therapies with agents that inhibit trogocytosis or checkpoint recycling may sustain therapeutic efficacy in hematologic and solid tumors.

#### Innate myeloid cell regulation

Mattei et al. (2022) demonstrated that dendritic cells and macrophages acquire tumor antigens via trogocytosis, influencing cross-presentation and anti-tumor immunity. Depending on the tumor context, this process can either amplify or suppress immune responses, highlighting the need for precise regulation of trogocytosis within the innate compartment.

#### NK cell desensitization

Miner et al. (2015) showed that NK cells acquire activating ligands from target cells via trogocytosis, paradoxically rendering them hyporesponsive. This ligand-mediated desensitization provides tumors with an immune evasion strategy. Therapeutic blockade of trogocytosis may preserve NK cell cytotoxicity in cancer and viral infections.

These mechanistic insights across immune compartments provide a conceptual foundation for understanding how trogocytosis contributes to immune dysregulation in systemic lupus erythematosus, particularly through checkpoint remodeling, epitope spreading, and genotype-informed modulation of tolerogenic axes.

### Signalling and molecular mediators of trogocytosis in SLE

**FIGURE 1:**
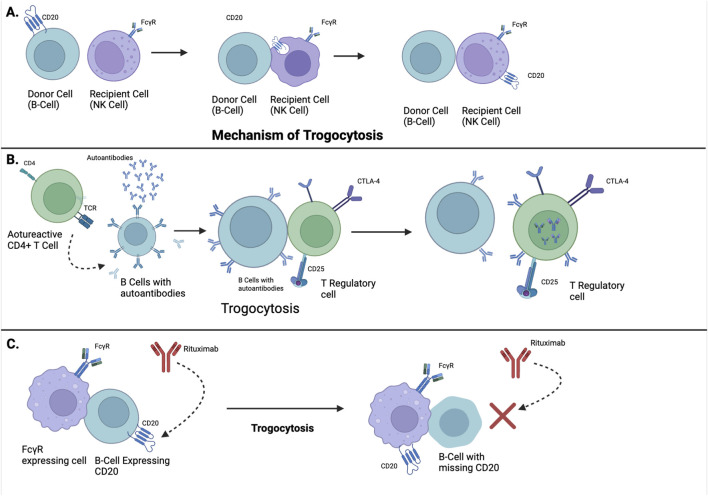
Mechanistic and Clinical Implications of Trogocytosis in Lupus Pathogenesis and Therapy. **(A)** Illustrates the process of trogocytosis, a phenomenon where immune cells physically extract membrane fragments, including surface molecules, from other cells during cell-cell contact. **(B)** Demonstrates how trogocytosis plays an immunomodulatory role in SLE, the autoreactive CD4^+^ T cells activate B cells to produce autoantibodies, which is a hallmark of lupus. Through trogocytosis, regs acquire molecules from B cells, enhancing their suppressive function. This helps limit the process of autoreactivity and dampen harmful autoimmunity. **(C)** Depicts therapeutic failure in lupus through trogocytosis. FcγR-expressing cells remove CD20-antibody complexes from B-cells, preventing Rituximab (an anti-CD20 monoclonal antibody) from binding. This antigen shaving makes B cells resistant to Rituximab, allowing autoreactive B cells to survive and continue disease activity.

Trogocytosis in systemic lupus erythematosus (SLE) is initiated at immunological synapses through antigen-specific receptor-ligand interactions, such as TCR–MHC II and BCR–antigen complexes. FcγR engagement on innate immune cells facilitates immune complex capture, particularly relevant in SLE’s autoantibody-rich milieu. Downstream signalling involves Src-family kinases (Lck, Fyn), PI3K-Akt, and MAPK pathways, promoting actin remodelling via WASp and Arp2/3. This orchestrates the transfer of immunomodulatory molecules (CD80, CD86, HLA-G), altering recipient cell function. Dysregulated trogocytosis facilitates epitope spreading, tolerance breakdown, and persistent autoreactivity, positioning it as a critical molecular mechanism and therapeutic target in SLE pathogenesis illustrated in Figure 1.

Future strategies that modulate trogocytic signaling or selectively redirect membrane transfer may offer novel avenues for restoring immune balance without broad immunosuppression.

### HLA-G mediated trogocytosis and immune checkpoint regulation in SLE

HLA-G, a tolerogenic non-classical MHC class I molecule, exerts immunomodulatory effects through its interaction with inhibitory receptors including ILT2 (LILRB1), ILT4 (LILRB2), and KIR2DL4These receptors are differentially expressed across immune subsets such as NK cells, CD8^+^ T cells, dendritic cells, and certain regulatory populations (LeMaoult et al., 2005). Trogocytosis enables the horizontal transfer of HLA-G from donor cells typically tolerogenic APCs or stromal elements to recipient effector cells, thereby transiently reprogramming their functional phenotype (Karczmarczyk et al., 2024). In SLE, this redistribution alters checkpoint engagement dynamics, potentially attenuating cytotoxicity via ILT2/KIR2DL4 signaling or impairing tolerogenic conditioning through ILT4 depletion (Tian et al., 2024; Wang et al., 2023). The net immunological outcome is context-dependent, influenced by receptor density, ligand affinity, and the genotype-driven variability in HLA-G expression and isoform distribution (Wang et al., 2023). Such trogocytic remodeling of checkpoint architecture may contribute to the fluctuating immune tolerance thresholds observed in lupus pathogenesis and offers a mechanistic basis for stratified immunotherapeutic interventions (Miyake and Karasuyama, 2021).

Trogocytosis mediates the intercellular redistribution of immunoregulatory molecules, including MHC complexes, CD80/CD86, and HLA-G thereby reshaping activation thresholds, promoting epitope spreading, and contributing to immune dysregulation in SLE. These effects are highly cell-type–specific, with distinct consequences for T cells, B cells, NK cells, and antigen-presenting cells. Zhou et al. demonstrated that CD4^+^ and CD8^+^ T cells acquire MHC-peptide complexes from antigen-presenting cells (APCs), altering their activation thresholds and promoting antigenic cross-presentation, which facilitates epitope spreading and amplification of autoimmune responses (Zhou et al., 2011). In systemic lupus erythematosus (SLE), sustained germinal centre interactions between B cells and T follicular helper (Tfh) cells enhance trogocytic transfer of co-stimulatory molecules, driving autoreactive B cell activation and pathogenic autoantibody production (Albanese et al., 2024). The transfer of HLA-G via trogocytosis may contribute to immune dysregulation in SLE through several mechanisms. First, the acquisition of HLA-G by cytotoxic CD8^+^ T cells from antigen-presenting cells could suppress their effector functions by engaging inhibitory receptors in an autocrine or paracrine manner, leading to reduced clearance of autoreactive immune cells (Mao et al., 2023). Second, B cells that acquire HLA-G through trogocytosis may experience altered signaling thresholds, impairing central and peripheral tolerance checkpoints and promoting the survival of autoreactive clones (Mao et al., 2023). Third, dendritic cells that lose surface HLA-G to neighboring lymphocytes may become hyperactivated due to diminished inhibitory feedback, resulting in enhanced antigen presentation and pro-inflammatory cytokine production (Zaborek-Łyczba et al., 2021). Collectively, these disruptions in immune cell crosstalk and regulatory balance may amplify the autoimmune cascade characteristic of SLE.

The non-classical MHC class I molecule HLA-G, a potent immunosuppressive checkpoint, can be redistributed among immune cells via trogocytosis (Rizzo et al., 2008). Upon acquisition, HLA-G is transiently expressed on the recipient cell surface, engaging inhibitory receptors such as ILT2, ILT4, and KIR2DL4. This modulates effector function by suppressing cytotoxicity and pro-inflammatory cytokine production, while promoting IL-10 secretion and regulatory phenotypes. However, in SLE, dysregulated HLA-G transfer may facilitate immune evasion by autoreactive lymphocytes and promote T cell exhaustion (Karczmarczyk et al., 2024).

Genetic polymorphisms affecting immune synapse integrity, MHC expression, and cytoskeletal regulation (e.g., PTPN22, LILRB1) may modulate trogocytosis efficiency and influence SLE heterogeneity (Reed et al., 2021). Thus, trogocytosis represents a critical immunoregulatory axis with dual pathogenic and therapeutic potential in lupus.

### Molecular specificity of trogocytosis in SLE

Beyond cell-type specificity, the nature of transferred molecules critically shapes downstream immune responses. Trogocytosis enables the rapid, contact-dependent exchange of membrane-bound immunomodulatory proteins such as CD80, CD86, and HLA-G between immune cells, thereby reshaping the functional landscape of recipient population (Gu et al., 2012). In systemic lupus erythematosus (SLE), this molecular redistribution can profoundly influence immune tolerance, epitope spreading, and autoreactivity. For instance, acquisition of CD80/CD86 by autoreactive B cells or effector T cells may enhance costimulatory potential and lower activation thresholds, while HLA-G transfer to cytotoxic subsets may transiently suppress effector functions via ILT2 and KIR2DL4 engagement (Bijl et al., 2001; Jacquier et al., 2020). Conversely, depletion of these molecules from tolerogenic antigen-presenting cells (APCs) or regulatory subsets may impair checkpoint signaling and promote immune escape (Ge et al., 2024).

These bidirectional effects underscore the complexity of trogocytosis as a cell-context–dependent amplifier of immune dysregulation. Moreover, the spatial and temporal dynamics of molecule transfer particularly during flare initiation or resolution remain poorly characterized but may hold diagnostic and therapeutic relevance (Wu et al., 2025). Integrating trogocytic profiles with single-cell transcriptomics and spatial proteomics could illuminate novel axes of immune modulation in lupus (Wang et al., 2025). These findings highlight the urgent need for high-resolution mapping of trogocytic exchanges across immune compartments in SLE.

### Immune cell-specific roles in trogocytosis and SLE

Trogocytosis is a rapid, contact-dependent process occurring at the immunological synapse, where immune cells exchange membrane fragments and surface proteins. Initially characterized by Dustin (2014), it is driven by stable receptor-ligand interactions that activate Src-family kinases (e.g., Lck, Fyn), initiating downstream PI3K-Akt and MAPK cascades. These signaling events converge on actin cytoskeletal remodeling via WASp and the Arp2/3 complex, enabling mechanical extraction of membrane patches enriched in MHC complexes, co-stimulatory ligands (CD80/CD86), adhesion molecules, and immunoregulatory proteins such as HLA-G. Transferred molecules may be transiently displayed or internalized, reprogramming recipient cell phenotype and function. Trogocytosis is a dynamic and selective process wherein immune cells actively extract and internalize fragments of plasma membrane including membrane-bound proteins such as HLA-G from adjacent cells during brief intercellular contact, resulting in the functional reprogramming of recipient cells by altering their surface receptor composition, signaling capacity, and immunological behavior (Zhai et al., 2023).

In SLE, trogocytosis contributes to pathogenesis by promoting epitope spreading, checkpoint redistribution, and altered antigen presentation. CD4^+^ T cells can acquire MHC class II and co-stimulatory molecules from APCs, potentially adopting antigen-presenting capabilities and amplifying autoreactive B cell activation (Choi and Morel, 2017). CD8^+^ T cells may capture MHC-peptide complexes, modulating cytotoxicity and contributing to tissue-specific autoimmunity (Zhang et al., 2007). B cells enhance their antigen-presenting capacity via trogocytosis from APCs or follicular dendritic cells, driving autoantibody production, hallmarks of SLE (Fahlquist-Hagert et al., 2023; Rastogi et al., 2022). NK cells, through Fcγ receptor–mediated engagement of lupus-associated immune complexes, may exhibit impaired apoptotic clearance and altered effector function (Liu et al., 2021). Dendritic cells transfer MHC and antigenic material to T cells, but dysregulated trogocytosis may hinder Treg induction and promote autoreactive T cell expansion (Nakayama et al., 2021; Schriek et al., 2022).

Collectively, these findings underscore the cell-type–specific consequences of trogocytosis in shaping immune dysregulation and disease progression in SLE. The accompanying diagram illustrates key molecular events and intercellular exchanges.

### Pathophysiological role of trogocytosis in systemic lupus erythematosus (SLE)

Emerging evidence implicates trogocytosis a contact-dependent intercellular transfer of membrane components as a critical, yet underrecognized, contributor to the immunopathogenesis of SLE through diverse mechanisms affecting antigen presentation, immune regulation, and effector responses.

Fcγ receptor (FcγR)-mediated trogocytosis induced by therapeutic monoclonal antibodies (e.g., epratuzumab, rituximab) selectively removes B cell surface molecules such as CD19, CD21, CD22, and CD79b without inducing cell death. This “antigenic shaving” impairs BCR signalling and antigen presentation, attenuating autoreactive responses. However, excessive modulation may diminish therapeutic efficacy by reducing target antigen density and inducing resistance (Taylor and Lindorfer, 2015).

Trogocytosis also facilitates “antigen cross-dressing,” wherein peptide–MHC complexes are transferred between dendritic cells, T cells, or apoptotic bodies, bypassing conventional antigen processing. This promotes the activation of autoreactive T cells and epitope spreading hallmarks of SLE progression (Schriek and Villadangos, 2023). Functionally, trogocytosis reprograms immune phenotypes. Regulatory T cells may acquire co-stimulatory molecules, altering suppressive capacity, while effector T and NK cells, acquiring inhibitory ligands (e.g., HLA-G, PD-L1), may undergo exhaustion. Conversely, co-stimulatory acquisition may potentiate hyperactivation (Chen S. et al., 2024).

Furthermore, in lupus nephritis and cutaneous SLE, FcγR-mediated trogocytosis of immune complexes amplifies inflammation, promotes cytokine release, and causes donor cell damage, fueling autoantigen release and perpetuating chronic autoimmunity. Thus, trogocytosis represents a central but underexplored mechanism in lupus immunopathology (Newling et al., 2020).

### Genetic modulation of trogocytosis and HLA-G pathways in systemic lupus erythematosus

Inter-individual variability in systemic lupus erythematosus (SLE) immunophenotypes and clinical outcomes is increasingly attributed to functional genetic polymorphisms affecting key regulatory pathways. Among these, genetic modulation of trogocytosis efficiency and human leukocyte antigen-G (HLA-G) expression plays a pivotal role in shaping immune cell interactions, tolerance induction, and disease pathogenesis.

#### HLA-G 3′untranslated region (3′UTR) polymorphisms

Polymorphic variants in the 3′UTR of the HLA-G gene, including the 14-bp insertion/deletion (INS/DEL), +3142 C>G, +3187 A>G, and +3196 C>G—impact mRNA post-transcriptional regulation by altering transcript stability and microRNA binding site availability. These variants form distinct haplotypes (UTR-1 to UTR-7) that correlate with differential levels of membrane-bound (mHLA-G) and soluble (sHLA-G) isoforms. For instance, UTR-1 is associated with reduced HLA-G expression due to destabilised transcripts, whereas UTR-5 and UTR-7 are linked to enhanced expression through increased transcript stability and decreased microRNA-mediated repression. These haplotypes thus influence the extent of immunomodulation mediated by HLA-G and its availability for trogocytic redistribution.

#### Regulatory polymorphisms in IL10 and IFN-I pathway genes

Variants in IL10 promoter regions (e.g., −1082 G>A, −592 C>A) modulate IL-10 transcriptional activity and consequently affect HLA-G upregulation, as IL-10 is a known inducer of HLA-G expression. Moreover, dysregulation of the type I interferon (IFN-I) axis, a hallmark of SLE, is driven by risk alleles in genes such as IRF5, STAT4, and IFIH1, which amplify IFN signalling. This hyperactivation may influence trogocytic activity by altering immune synapse architecture, receptor clustering, and cytoskeletal remodelling, thereby modulating the efficiency of membrane fragment transfer and immune cell reprogramming.

#### Implications for immunopathological stratification and therapy

The combinatorial effects of polymorphisms in HLA-G, IL10, and IFN-I pathway genes provide a molecular framework for patient stratification in SLE. Individuals harbouring genotypes associated with high HLA-G expression may exhibit enhanced immunosuppressive signalling and increased susceptibility to immune evasion. Conversely, those with low-expressing haplotypes may display impaired tolerance checkpoints and augmented inflammatory responses. These genotype-dependent profiles suggest the potential utility of integrated immunogenomic signatures in guiding personalised therapeutic strategies, particularly interventions targeting trogocytosis, immune checkpoint modulation, and HLA-G–mediated tolerance in lupus.

### Trogocytosis-mediated modulation of HLA-G in SLE: a genotype-informed immunoregulatory axis

Emerging evidence identifies trogocytosis as a pivotal immunoregulatory mechanism in systemic lupus erythematosus (SLE). This rapid, contact-dependent process enables selective transfer of membrane-bound molecules including MHC complexes and immune checkpoints between immune cells, thereby modulating cell phenotype, activation thresholds, and antigen presentation (Agbakwuru and Wetzel, 2024). Unlike classical phagocytosis, trogocytosis preserves cell viability while reshaping surface receptor landscapes, supporting dynamic immune regulation. Notably, HLA-G redistribution via trogocytosis alters checkpoint engagement and tolerogenic signaling across immune subsets (Kim et al., 2025). Pharmacogenomic data further implicate polymorphisms in immune regulatory loci as modulators of trogocytic efficiency and specificity, highlighting the potential for genotype-informed therapeutic strategies targeting the HLA-G axis in SLE (Agbakwuru and Wetzel, 2024).

### Therapeutic implications of trogocytosis in systemic lupus erythematosus

Emerging evidence implicates trogocytosis not only as a contributor to immune dysregulation in systemic lupus erythematosus (SLE) but also as a promising therapeutic target. While traditionally viewed as a mechanism facilitating antigen spreading and aberrant intercellular signalling, recent insights reveal that controlled manipulation of trogocytosis may enable selective immunomodulation without inducing systemic immunosuppression (Miyake and Karasuyama, 2021).

#### Antibody-based modulation of trogocytosis

Innovations in antibody engineering have enabled the development of bispecific antibodies designed to exploit Fcγ receptor (FcγR)-mediated trogocytosis for therapeutic purposes. Wang et al. have highlighted bispecific constructs targeting B cell–specific markers such as CD19 and CD22, combined with Fc domains capable of engaging FcγRs on effector cells like macrophages and dendritic cells. This dual targeting facilitates selective extraction of activation-associated surface receptors (e.g., CD80, CD86, BCR components) from autoreactive B cells through trogocytic or antibody-dependent cellular phagocytic mechanism, —without inducing broad B cell depletion (Ramezani et al., 2023).

#### Functional reprogramming of immune cells

Beyond B-cell targeting, trogocytosis may be therapeutically leveraged to modulate effector cell immunophenotypes. By facilitating the acquisition or removal of regulatory molecules such as HLA-G or PD-L1, trogocytosis can alter T cell activation thresholds, promote regulatory T cell–like phenotypes, or disrupt pathogenic immune cell interactions. Such context-specific modulation could restore immune tolerance in SLE while minimising systemic toxicity (Contini et al., 2020).

Despite growing recognition of trogocytosis as a dynamic modulator of immune cell function, a comprehensive synthesis of its role in SLE pathogenesis remains lacking. Current literature highlights cell-specific consequences of trogocytic exchange such as altered antigen presentation by dendritic cells, checkpoint remodeling in T and NK cells, and epitope spreading via B cell mediated transfer but these findings are often siloed and mechanistically under-integrated (Fahlquist-Hagert et al., 2023). Moreover, the temporal and spatial dynamics of trogocytosis in lupus flares, tolerance breakdown, and tissue-specific immune infiltration remain poorly characterized. Therapeutically, targeting trogocytosis offers novel avenues for modulating immune thresholds, restoring checkpoint balance, and reprogramming autoreactive circuits (Chen Y. et al., 2024). However, translating these insights into clinical interventions requires deeper mechanistic mapping, integration of genotype-informed variability, and validation across diverse SLE cohorts. A unified framework that links trogocytic mechanisms to disease progression and therapeutic responsiveness could significantly advance precision immunomodulation in lupus (Tian et al., 2025).

### Challenges and prospects

Despite its therapeutic potential, the dualistic nature of trogocytosis as both a facilitator of autoimmunity and a mechanism of immune regulation necessitates precision targeting. Genetic and cellular heterogeneity among patients may influence the direction and impact of trogocytic events. A deeper understanding of the molecular determinants governing trogocytosis, including cytoskeletal regulation, receptor clustering, and synapse formation, is essential for optimising this strategy (Miyake and Karasuyama, 2021; Ramezani et al., 2023).

### Discussion and future direction

The emerging role of trogocytosis in the immunopathogenesis of systemic lupus erythematosus (SLE) highlights the intricate dynamics of intercellular communication in autoimmunity. Once considered a secondary immunological phenomenon, trogocytosis is now recognised as a pivotal process that can exert both immunosuppressive and immunostimulatory effects, depending on the molecular cargo transferred and the immunological context. In SLE, the hyperactivation of B and T lymphocytes, combined with aberrant antigen-presenting cell (APC) function, establishes a milieu conducive to pathological trogocytic interactions.

Comparative studies have demonstrated that HLA-G expression is significantly altered in patients with systemic lupus erythematosus (SLE) compared to healthy individuals. In healthy controls, CD14^+^ monocytes and dendritic cells exhibit stable surface HLA-G expression, which can be upregulated by cytokines such as IFN-γ and IL-10 (Monsiváis-Urenda et al., 2011). In contrast, SLE patients show reduced surface HLA-G levels and impaired cytokine responsiveness, suggesting a breakdown in tolerogenic signaling. Additionally, circulating levels of soluble HLA-G (sHLA-G) are elevated in SLE, potentially reflecting compensatory immune regulation or dysregulated isoform expression linked to HLA-G 14-bp I/D polymorphisms (Jucaud et al., 2016).

Emerging evidence also implicates trogocytosis in autoimmune pathogenesis, where immune cells actively exchange membrane fragments and surface proteins. This process has been observed to facilitate immune evasion and plasticity, and in autoimmune contexts, may contribute to the mislocalization or functional alteration of regulatory molecules like HLA-G. These findings support the hypothesis that defective trogocytosis-mediated HLA-G transfer may exacerbate immune imbalance in SLE.

Through the acquisition of MHC-peptide complexes, co-stimulatory ligands, and immunomodulatory molecules such as HLA-G, immune cells may undergo phenotypic reprogramming. This modulates antigen presentation and contributes to epitope spreading, immune effector cell exhaustion, or inappropriate activation core features of lupus pathogenesis. Particularly, HLA-G, typically associated with immune tolerance, may paradoxically facilitate immune escape when aberrantly expressed or redistributed via trogocytosis. Polymorphisms in the HLA-G 3′untranslated region (UTR), IL10 promoter, and type I interferon pathway genes (e.g., STAT4, IRF5) influence trogocytic efficiency and HLA-G expression, potentially accounting for inter-individual variability in disease severity and treatment response.

From a therapeutic perspective, engineered bispecific antibodies targeting trogocytosis pathways have emerged as a precision strategy to modulate autoreactive B cells without complete depletion, preserving immune competence. Such approaches offer a targeted alternative to broad immunosuppressive therapies. However, the dual nature of trogocytosis necessitates careful calibration to prevent exacerbation of autoimmunity.

Taken together, these findings support a unified model in which trogocytosis-mediated redistribution of HLA-G contributes to immune imbalance in systemic lupus erythematosus (SLE) (Martín-Villa et al., 2022). Under physiological conditions, HLA-G is selectively transferred from tolerogenic antigen-presenting cells to effector lymphocytes, promoting immune quiescence through inhibitory signaling (Contini et al., 2020). In SLE, however, this intercellular transfer is disrupted or misdirected: CD8^+^ T cells may receive insufficient HLA-G to suppress autoreactivity; B cells may acquire aberrant HLA-G signals that bypass tolerance checkpoints; and dendritic cells, depleted of surface HLA-G, may become hyperactivated, enhancing antigen presentation and inflammatory cytokine release (Chen and Tsokos, 2021). This dysfunctional redistribution creates a self-amplifying loop of immune activation and loss of tolerance, positioning trogocytosis not as a passive exchange but as a critical regulatory mechanism whose breakdown may drive autoimmune pathology in SLE.

In summary, trogocytosis represents a context-dependent, genetically modulated process that is integral to SLE pathophysiology and holds promise as a novel therapeutic axis. Future studies should aim to refine their clinical applications while delineating their mechanistic underpinnings.
